# Wavelength-Tunable, Low-Angular Dispersion, and Narrowband Thermal Emitters by Incorporating Ge_2_Sb_2_Te_5_ Layer into Grating-Assisted Multilayered Structures

**DOI:** 10.1021/acsami.6c06818

**Published:** 2026-06-22

**Authors:** Yuan-Wei Chang, Po-Wei Ho, Hui-Hsin Hsiao

**Affiliations:** † Department of Engineering Science and Ocean Engineering, 33561 National Taiwan University, Taipei 10617, Taiwan; ‡ Institute of Electro-Optical Engineering, 34879 National Taiwan Normal University, Taipei 11677, Taiwan; § Graduate Institute of Photonics and Optoelectronics, National Taiwan University, Taipei 10617, Taiwan

**Keywords:** thermal emitters, phase-change materials, blackbody radiation, localized surface plasmons, mid-infrared light sources

## Abstract

High-quality-factor (*Q*-factor) mid-infrared (MIR) light sources with spectral tunability and minimal angular dependence are highly desirable for practical sensing applications. In this work, a continuously tunable, low-angular dispersion, and narrowband thermal emitter is realized by incorporating a Ge_2_Sb_2_Te_5_ (GST) layer into the grating-assisted distributed Bragg reflector (DBR)-like structure. The multilayer back reflector is designed to achieve high reflectance over a broad DBR-like stopband, while simultaneously avoiding direct contact with the metallic back reflector, as in conventional metal–dielectric–metal (MDM) structures. Upon illumination with transverse-magnetic (TM)-polarized light, the hybrid structure is found to support a hybridized localized surface plasmon (h-LSP) with a *Q*-factor of 30.64 in simulations (in contrast to the LSP mode in MDM structures with a *Q*-factor of 6.17) due to the reduction of ohmic loss. Meanwhile, the h-LSP mode shows a similar geometric dependence on grating width and exhibits a negligible wavelength variation under varying oblique incidence angles. Samples with three grating widths of 1.4 μm, 1.7 μm, and 2.0 μm were fabricated to support the h-LSP mode at distinct wavelengths. Upon thermal annealing, both the measured reflection and emission spectra of these samples exhibit a continuous redshift, with an average shift of 140 nm corresponding to an approximately 40% crystalline fraction of GST. Meanwhile, the Q-factors of the emission peaks remain above 8.70 for *w* = 1.4 μm, 12.72 for *w* = 1.7 μm, and 15.68 for *w* = 2.0 μm. Such a tunable, narrowband, and small angular-dependent MIR light source is promising for enhancing the accuracy in the discrimination of molecular fingerprints.

## Graphical abstract

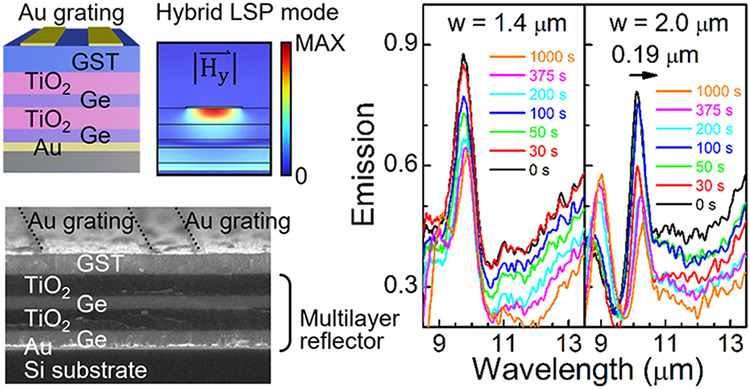

## 1 Introduction

Wavelength-tunable mid-infrared (MIR) emitters are essential devices in gas sensing, infrared imaging, solar thermophotovoltaics, and adaptive camouflage.^1–6^ Among these applications, emitters with narrowband, nonspatially dispersion, and spectral selective are highly desirable for the light source in molecular sensing, as the absorption line widths of most target chemical substances are narrow.^7–9^ To tailor thermal radiation into well-controlled and tunable emission at a single wavelength, a large variety of thermal emitters have been demonstrated based on the designs of photonic crystals,^10–13^ surface plasmon polaritons (SPPs),^14–16^ localized surface plasmons (LSPs),^17–21^ gap-plasmon modes,^22–25^ and Tamm plasmon polaritons (TPPs).^26–30^ For example, the gap-plasmon mode (also referred to as the LSP mode) in metal–dielectric–metal (MDM) sandwich structures can be readily tuned by adjusting the top antenna line width, the intrinsic refractive index of the dielectric material, or the spacer thickness.^17–20^ Due to its localized resonant nature, the gap-plasmon mode shows minimal angular dependence in its emission wavelength, thereby improving the reliability of sensing applications. However, the intrinsic ohmic loss of metallic components leads to broad emission spectra in LSP-based thermal emitters. On the other hand, TPP modes, exciting at the interface between a metallic mirror and a distributed Bragg reflector (DBR), exhibit a high-quality factor (Q-factor) resonant profile.^30,31^ As TPP is determined by the composed multilayers of the DBR especially the thickness of the cavity, most of the TPP-based thermal emitters provide narrowband emission at one single peak wavelength and can hardly be used for multicompound sensing.^32^

In addition to static spectral selectivity achieved through geometric control, it is desirable to realize continuously tunable emissivity by incorporating dynamically reconfigurable materials into device designs, such as liquid crystals,^33–35^ graphene,^36,37^ microelectromechanical systems,^38^ and acoustic-optical interactions.^39^ However, many of these approaches suffer from significant drawbacks, including the need for moving parts, limited spatial resolution, and slow response time.^40^ In contrast, phase-change materials (PCMs) such as VO_2_,^41–43^ Ge_2_Sb_2_Te_5_ (GST),^40^ and In_3_SbTe_2_ (IST)^44^ offer advantages such as fast switching speeds, low optical losses in the infrared region, and a high refractive index contrast. GST stands out for its unique optical properties in the MIR range: amorphous GST (aGST) acts as a lossless dielectric, while crystalline GST (cGST) functions as a low-loss dielectric, enabling tunable narrowband thermal emission.^40^ Unlike VO_2_-based meta-devices, the nonvolatile GST retains its optical properties until reprogrammed and exhibits excellent thermal stability at room temperature after the phase transition.^45–47^ With appropriate thermal treatment, the crystallinity level of GST can be precisely controlled to achieve the desired optical characteristics.

For example, many early studies have demonstrated tunable thermal emission by incorporating a GST layer into simple layered structures, including gold (Au)/GST bilayers,^48^ aluminum/GST bilayers,^49^ and chromium/GST/Au multilayers.^49^ These approaches enable a large emission spectral shift exceeding 3 μm by switching the GST layer between amorphous and crystalline states. However, the emission bandwidth is also broad with a full width at half-maximum (FWHM) close to 3 μm. Subsequently, various MDM structures incorporating a GST spacer sandwiched between metallic layers have been investigated. In these designs, the FWHM of the emission peaks is reduced to below 1 μm, although the phase-change-induced emission wavelength shift is correspondingly smaller, typically around 1 μm or less.^21,50–52^ In addition, the magnetic resonances in GST nanodisks on an Au film have been experimentally demonstrated to achieve a spectral shift of around 2 μm, but the emission bandwidth becomes noticeably broader because GST becomes more lossy at higher crystalline fractions. On the other hand, combining a GST layer with a DBR has enabled tunable narrowband MIR thermal emitters with a high *Q*-factor of up to 172.^53^ Later, a Fabry–Perot mode formed by incorporating a GST cavity layer between two DBRs was demonstrated, achieving a spectral tunability of about 300 nm while maintaining a *Q*-factor of 70–90 between the amorphous and crystalline states.^54^ However, this work only demonstrates a transmission filter and does not exhibit emission properties.

In this paper, we design a continuously tunable high-Q plasmonic thermal emitters (PTE) by incorporating top Au gratings, a GST spacer, and a multilayer back reflector (Figure 1a). The back reflector is constructed by alternately stacking germanium/titanium dioxide (Ge/TiO_2_) layers on a bottom Au film. Under transverse-magnetic (TM)-polarized illumination, such a hybrid structure sustains a hybrid LSP (h-LSP) mode which preserves the same geometric tunability of the LSP (gap plasmon) mode in a conventional MDM configuration, while exhibits a significantly narrower resonant line width. A systematic numerical study is conducted to investigate the optical properties of the h-LSP mode, and its dispersion relation is further analyzed by examining the reflection spectra under varying oblique incidence angles. By fabricating samples with different grating widths, the h-LSP mode is shown to produce high-*Q* radiation peaks at distinct wavelengths upon thermal excitation of the device. Thermal annealing of the devices at 160 °C on a hot plate for varying durations induces a continuous redshift of 140 nm in the radiation peaks, while the *Q*-factor remains above 8.70 at an approximately 40% crystalline fraction of the GST layer. The flexibility in controlling the high-*Q* emission via the grating width, together with fine-tuning the emission wavelength through the phase transition of the GST layer, enables precise control of the emission wavelength. Moreover, the minimal angular dependence of the h-LSP mode enhances the stability of the emission wavelength, thereby improving accuracy in sensing and molecular fingerprint analysis applications.

**Figure 1:**
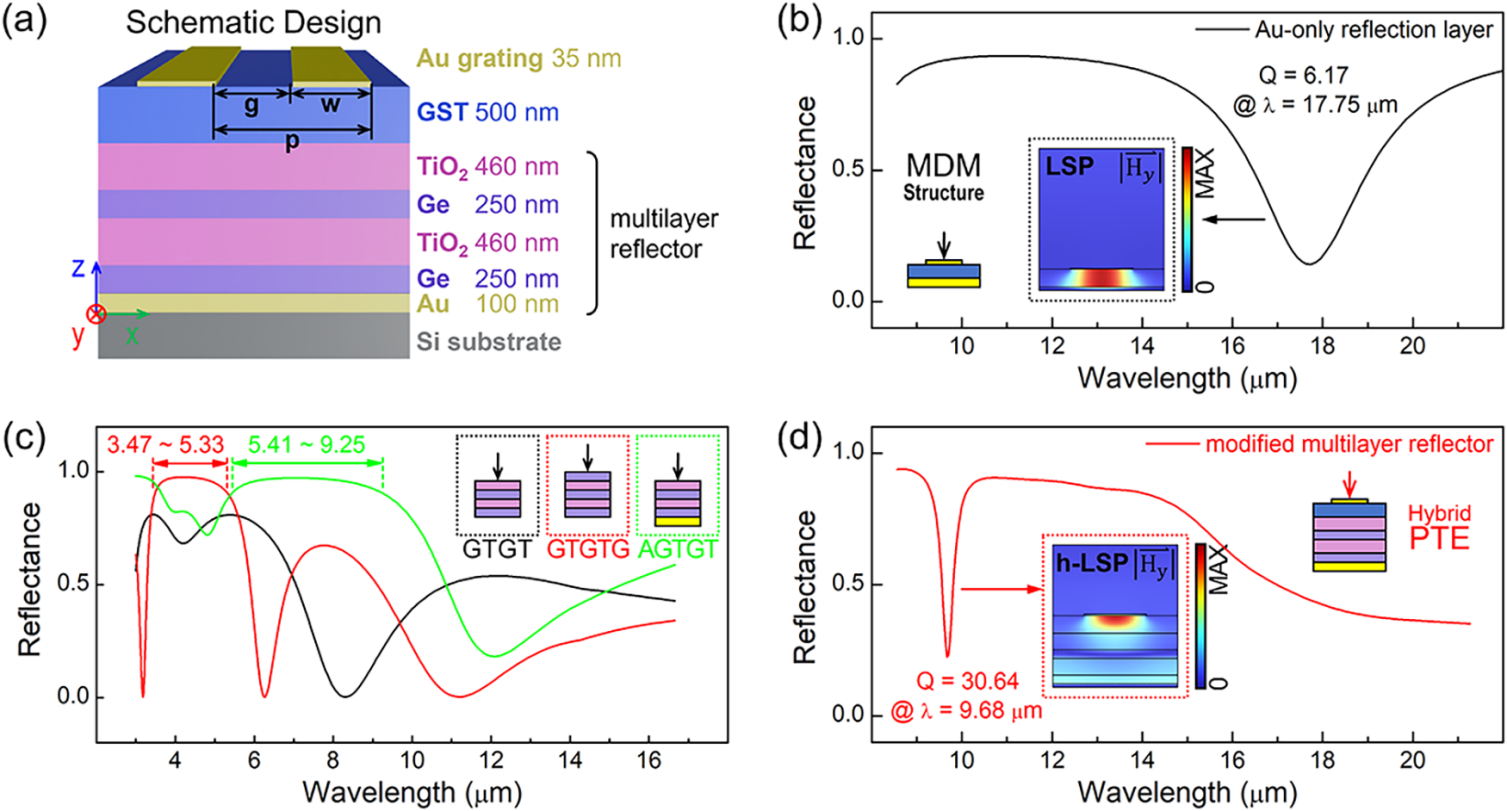
(a) The schematic design of hybrid PTEs consisting of the modified multilayer reflector, the GST spacer, and the top Au gratings. (b) Simulated reflectance spectra of the MDM structure with a top grating width (*w*) of 1.75 μm and period (*p*) of 3.5 μm under *x-*polarized light illumination. The insets show the near-field distributions of the LSP mode at 17.75 μm. (c) Simulated reflectance spectra of different reflectors, four-layered Ge/TiO_2_ DBRs (denoted as the GTGT structure, black curve), five-layered Ge/TiO_2_ DBRs (denoted as the GTGTG structure, red curve), four-layered Ge/TiO_2_ DBRs on a gold film (denoted as the AGTGT structure, green curve), where G, T, and A represent Ge, TiO_2_, and Au, respectively. (d) Simulated reflectance spectra of our hybrid PTE incorporating the GST spacer and the modified multilayer reflector. The inset shows the near-field distribution of the h-LSP mode at 9.68 μm under *x*-polarized light incidence.

## 2 Structures and Methods

### 2.1 Design and Simulations

All of the simulations were performed by commercial software COMSOL Multiphysics software (COMSOL Inc.). The two-dimensional (2D) model was constructed in the *x*–*z* plane, as illustrated in Figure 1a. Floquet periodic boundary conditions were applied along the lateral directions of the unit cell to simulate the reflection spectra and near-field distributions of the periodic structure. TM-polarized light was incident from above the structure. The refractive index of gold, Ge, TiO_2_, and silicon substrates were obtained from the literature.^55^

The effective permittivity of the GST with different levels of crystallinity is evaluated using the Lorentz–Lorenz equation:^56^

1 $$\frac{\varepsilon_{X}-1}{\varepsilon_{X}+2}=\left(1-X\right)\frac{\varepsilon_{a}-1}{\varepsilon_{a}+2}+X\frac{\varepsilon_{c}-1}{\varepsilon_{c}+2}$$

where ε_a_, ε_c_, and ε_X_ refer to the permittivity of aGST, cGST, and GST with *X* level of crystallization, respectively. *X* represents the level of crystallization, ranging from 0 to 1. *X* = 0 means 100% amorphous and 0% crystalline; on the contrary, *X* = 1 means 0% amorphous and 100% crystalline. The refractive index and extinction coefficient of aGST and cGST used in our numerical model are displayed in Figure S1.

### 2.2 Fabrication and Heat Treatment

The entire fabrication process of the hybrid PTE is illustrated in Figure S2. First, the sliced 1.5 cm × 1.5 cm Si substrate was cleaned by the deionized water, acetone, and IPA for 3 min, and the cleaning sequence was repeated three times in an ultrasonic cleaner. On the cleaned Si substrate, a 100-nm-thick Au with a 5 nm Ti adhesive layer was deposited by thermal evaporation. Then, two pairs of alternating Ge and TiO_2_ multilayers were deposited on top of the Au layer by thermal evaporation, with thicknesses of 250 nm for the Ge layers and 460 nm for the TiO_2_ layers, respectively. Subsequently, a 500-nm*-*thick GST layer was sputtered onto the multilayer reflector at a power of 150 W. Finally, the grating pattern was defined by applying the negative photoresist NR9-1000PY and performing a photolithography process. A 35-nm*-*thick Au layer was then deposited by thermal evaporation with the 5 nm Ti adhesive layer as well, followed by a lift-off process to complete the fabrication of the 1D Au grating. Table 1 summarizes the designed and measured thicknesses of each layer in the device, along with their corresponding deviations.

**Table 1:** Designed and Measured Thicknesses of the Hybrid PTE Devices

materials	designed thickness (nm)	measured thickness (nm)	error (%)
Au grating	35	36.1 ± 1.3	3.14
GST cavity	500	520.4 ± 8.5	3.40
TiO_2_ (upper)	460	486.7 ± 10.0	5.81
Ge (upper)	250	281.6 ± 11.4	12.66
TiO_2_ (lower)	460	484.2 ± 2.8	5.26
Ge (lower)	250	276.9 ± 11.0	10.74
Au	100	95.8 ± 4.5	4.20

The thermal annealing treatment was conducted by placing the samples on a hot plate at 160 °C for different durations, ranging from 30 s, 50 s, 100 s, 200 s, and 350 to 1000 s, to induce varying degrees of crystallinity. To investigate the optical behavior of the tunable PTEs, reflectance and emission measurements were first performed on the as-fabricated samples. After the PTEs underwent heat treatment, the measurements were repeated to evaluate the influence of the GST phase transition.

### 2.3 Examination and Verification

The microstructures of the tunable PTE devices were primarily characterized using a scanning electron microscope (SEM, model: JEOL-6500). The fabricated samples were sectioned to obtain cross-sectional images and verify the thickness of each deposited layer. The geometry of the 1D Au grating was also examined using plan-view SEM images. The crystallinity variation associated with the phase change of the GST layer was characterized by grazing-incidence X-ray diffraction (GIXRD, model: Bruker D8 Advance Plus). In GIXRD, the X-rays are directed onto a sample at a very low incident angle, which causes the X-rays to interact with only the top few nanometers of the material. This results in a diffraction pattern that is highly sensitive to the crystallographic properties of the surface region, which is highly suitable for observing the crystallinity of GST in this study. The optical behavior of the tunable PTEs before and after the phase transition was characterized using Fourier transform infrared spectroscopy (FTIR, model: Vortex-70).

## 3 Results and Discussion

Figure 1a schematically illustrates the hybrid PTE design consisted of three parts: the multilayer reflector (Au 100 nm/Ge 250 nm/TiO_2_ 460 nm/Ge 250 nm/TiO_2_ 460 nm), the 500-nm*-*thick GST layer, and the top Au grating with a thickness of 35 nm. Beneath the proposed structure is a silicon substrate. The top Au stripes have a width of *w* separated by a gap distance of *g* that determines the periodicity (*p*) of the grating. We first analyzed the resonant modes in a conventional PTE design consisting of a 35-nm-thick Au top grating, a 500 nm GST spacer, and a 100 nm Au back reflector (Figure 1b) (see computational details in Sections 2 and S1). Figure 1b shows the simulated reflection spectra of the MDM structure with a top grating width of 1.75 μm and a period of 3.5 μm. Under *x*-polarized light illumination, a resonant dip is observed at λ = 17.75 μm. The corresponding magnetic field distribution exhibits the characteristic resonant feature of the gap-plasmon mode (also known as the LSP mode), showing a standing-wave-like pattern confined beneath the gratings within the GST spacer (inset in Figure 1b). Due to the intrinsic metallic loss in the conventional MDM structure, the LSP mode shows a broad resonant width with a *Q*-factor of 6.17. The *Q*-factor is defined as the ratio of the resonant frequency to the bandwidth.

To reduce the effect of ohmic loss, we thus modified the Au back reflector by introducing two pairs of alternating stacked dielectric layers on top of the Au layer. Figure 1c displays the simulated reflectance spectra for the four-layered distributed Bragg reflectors (DBRs) stacked by alternative high and low refractive index materials of Ge and TiO_2_ designed at a central wavelength of 4 μm, corresponding to a quarter-wave optical thickness of 250 nm for Ge and 460 nm for TiO_2_. One can observe that the constructive interference of the four-layered DBRs (denoted as GTGT) is still not sufficient to produce high reflectance at the designed spectral band and shows maximal reflectance of 0.81 around λ = 4 μm (black curve). By introducing an additional Ge layer, the fived-layered DBRs (denoted as GTGTG) show a high reflectance stopband (>0.9) within the wavelength range of 3.47–5.33 μm (red curve in Figure 1c). Interestingly, when we combined the four-layered DBR on an Au-reflector (denoted as AGTGT), the reflectance of the modified multilayer reflector is larger than 0.9 within a broader spectral range of 5.41 μm and 9.25 μm (green curve). The measured reflection of the AGTGT structure is displayed in Figure S3.

We thus utilized this modified multilayer reflector to replace the Au back reflector and form our hybrid PTE structure as illustrated in Figure 1a. The replacement aims to reduce the ohmic loss induced by the bottom Au reflector. The high reflectivity of the multilayer reflector enables the formation of a confined mode beneath the top grating within the GST cavity while simultaneously preventing direct contact with the bottom metallic layer, thereby improving the *Q*-factor of the resonant mode. The new hybrid structure shows a resonant dip at λ = 9.68 μm under the illumination of *x*-polarized light (Figure 1d). We identified this resonant mode as the hybridized LSP mode (denoted as h-LSP) because its optical property is similar to those of the LSP mode supported by the MDM structure but with a much higher *Q*-factor of 30.64. The near-field distribution also reveals a strong magnetic field concentrated beneath the grating width and within the GST spacer (the inset of Figure 1d). To systematically investigate the geometry dependence of this resonance mode in the hybrid PTE structure, Figure 2 shows the simulated absorption spectra under the variation of grating width (*w*), gap distance (*g*), and grating periodicity (*p*), respectively. Since light transmitted through the device is eliminated by the Au ground plane, the absorption spectra were obtained from the energy conservation law of *A* = 1 – *R*, where *A* and *R* represent absorption and reflection, respectively. Figure 2a shows the absorption spectra when *w* is varied under a constant *g* of 1 μm. One can observe that the h-LSP mode redshifts from λ = 9.03 μm to λ = 9.86 μm when *w* is increased from 1 to 2.5 μm. As the variation of *w* will also change *p* under a constant grating gap *g* (i.e., *p* = *w* + *g*), we also simulated the absorption spectra with a fixed *p* = 3.5 μm and varied *w* to distinguish the effect of grating width and the overall grating periodicity. As shown in Figure 2b, the h-LSP resonance still exhibits a similar redshift trend from λ = 8.87 μm to λ = 9.86 μm when enlarging *w* from 1 μm to 2.5 μm. In addition, when *w* is kept at 1 μm, the h-LSP resonance only slightly blueshifts as the gap size increases. This gives us a clue to the dependence of the h-LSP mode on the grating width, which can be expressed as follows:

2 $$m\lambda_{h‐LSP}=2n_{eff,h‐LSP}\times w$$

where *m* is an integer, λ_h‑LSP_ is the resonant wavelength of the h-LSP mode, *n*_eff,h‑LSP_ is the effective refractive index of the h-LSP mode, and *w* is the grating width. In addition, to ensure the emission wavelength of the PTE remains stable and narrowband across different radiation angles, we also simulated the absorption spectra for the sample with *w* = 1.75 μm and *p* = 3.5 μm by varying the oblique incidence from 0° to 60° under TM-polarized illumination. As shown in Figure 2d, The h-LSP mode exhibits only a slight blueshift of 0.04 μm as the oblique incidence angle varies from 0° to 50°, demonstrating its weak angular dependence.

**Figure 2:**
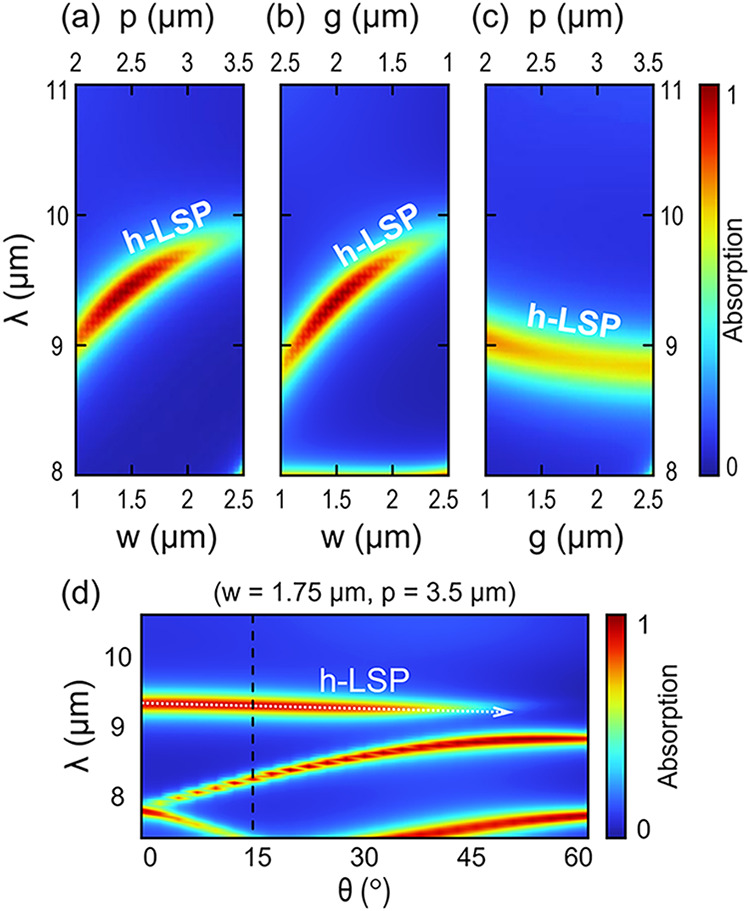
Geometric dependence of the h-LSP mode. Under TM-polarized incidence, the simulated absorption spectra of the designed PTEs for (a) varying *w* with *g* fixed at 1 μm, (b) varying *w* with *p* fixed at 3.5 μm, and (c) varying *g* with *w* fixed at 1.0 μm. (d) The simulated absorption spectra for the sample with *w* = 1.75 μm and *p* = 3.5 μm by varying the oblique incident angle from 0° to 60°.

The hybrid PTE structures were then fabricated through a series of thin-film deposition steps, followed by conventional photolithography to define the top grating patterns (see fabrication details in Sections 2 and S2). We fabricated the hybrid PTE structures with various top Au-grating widths of 1.4 μm, 1.7 μm, and 2.0 μm under the same grating periodicity of 3.5 μm. The top views of SEM images for the fabricated samples are displayed in Figure 3a–c. The thicknesses of the deposited nanolayers were also examined by the cross-sectional view of the SEM images as shown in Figure 3d, and the measured thickness of each layer is summarized in Table 1. The optical characterization of the fabricated samples was then measured by a Bruker Vertex 70 FTIR spectrometer to measure the reflection spectra, the angular dispersion relation, and the thermal radiation.

**Figure 3:**
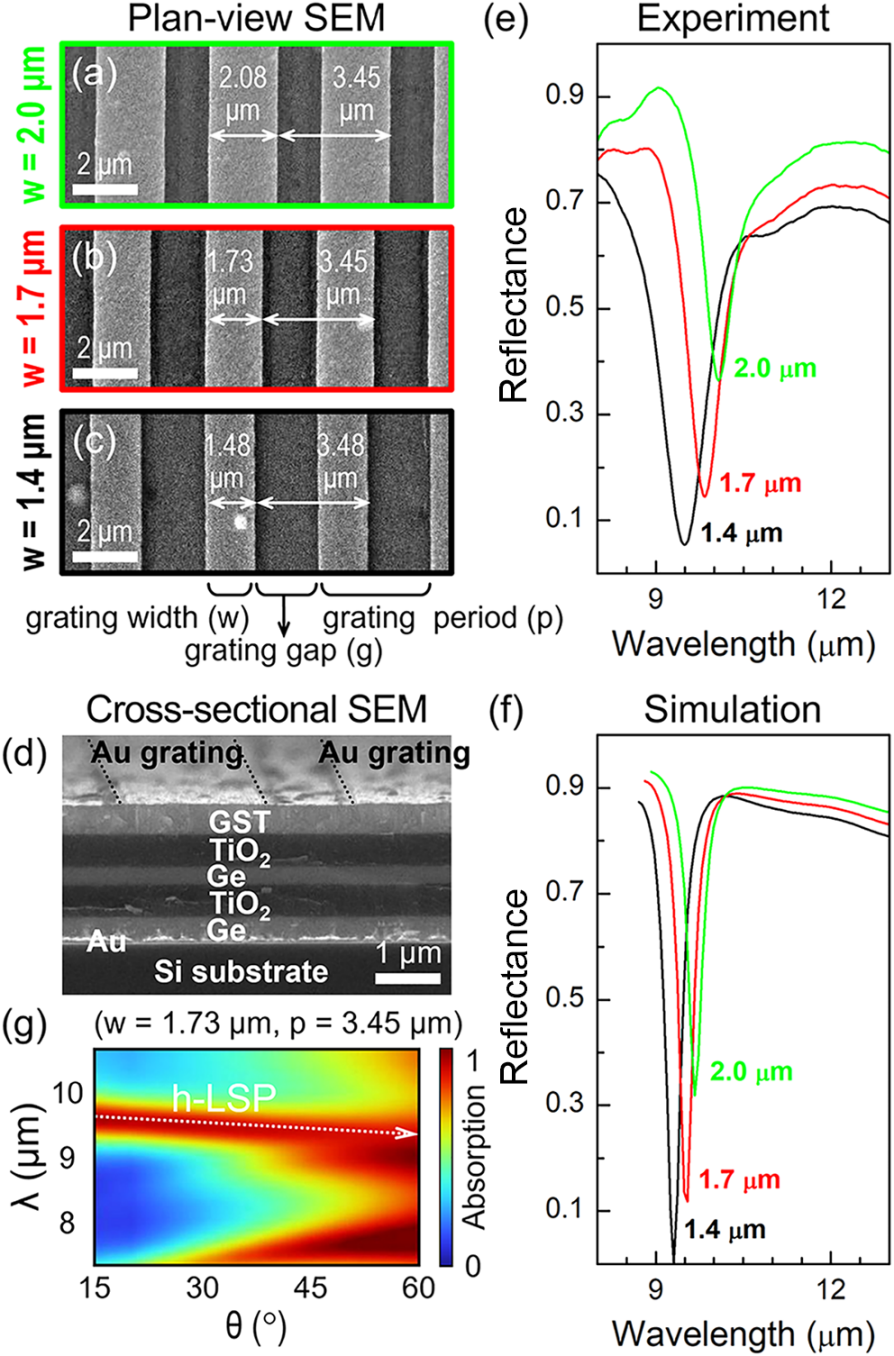
Plan-view SEM images for samples with the grating width of (a) 2.08 μm, (b) 1.73 μm, and (c) 1.48 μm, respectively, and a fixed periodicity of 3.45 μm. (d) The cross-sectional SEM image of the tunable PTE structure. The corresponding (e) measured and (f) simulated reflectance spectra for samples with different grating widths under oblique incidence of TM-polarized light (θ = 15°). (g) The measured absorption spectra as a function of incident angles varying from 15° to 60° for w = 1.73 μm and *p* = 3.45 μm.

Figure 3e,f shows the measured and simulated reflection spectra for samples with *w* = 1.4 μm, 1.7 μm, and 2.0 μm, respectively, under the oblique incidence of TM-polarized light with θ = 15°. The h-LSP mode in the measured spectra is observed at λ = 9.50 μm for *w* = 1.4 μm and gradually redshifts to λ = 9.86 μm and λ = 10.09 μm as *w* increases to 1.7 and 2.0 μm, respectively. Meanwhile, the corresponding *Q*-factor from the measured reflection is 8.81 for *w* = 1.4 μm, increasing to 12.88 and 18.63 for *w* = 1.7 and 2.0 μm, respectively. The simulation results show good agreement with the experimental data, exhibiting a similar redshift trend of the h-LSP mode accompanied by a gradual decrease in resonance depth as *w* increases. Without the inevitable surface roughness and dimension variation for the fabricated sample, the *Q*-factor of the h-LSP mode in the simulated spectra is expected to be higher, reaching 24.7, 25.1, and 23.7 for *w* = 1.4 μm, 1.7 μm, and 2.0 μm, respectively. In addition, to examine the angular dependence of the h-LSP mode, we also measured the absorption spectra at oblique incidence angles ranging from 15° to 60° in 5° increments. The measured absorption spectra of the sample with *w* = 1.73 μm and *p* = 3.45 μm exhibit a blueshift of 0.172 μm as the oblique incidence angle varies from 15° to 35°, while another mode at a shorter wavelength begins to merge with the h-LSP mode when the angle exceeds 35° (see detailed analysis of the resonance wavelength versus the oblique incidence angle for three samples in Section S4).

The fabricated samples were then treated by thermal annealing at 160 °C on a hot plate. The thermal annealing temperature was pretested to ensure that GST underwent a relatively slow phase transition process, allowing better control over the different stages of the transition.^57,58^ The phase transition of GST in the hybrid PTE device was verified by glazing-incidence X-ray diffraction (GIXRD). As shown in Figure S6a, due to the presence of the top Au gratings, strong Au (111) and (200) signals were also observed. Figure S6b–d represents the signals of GST (111), (200), and (220) of the face-centered cubic (FCC) phase, respectively. All of these signals gradually increased after 50 *s* of annealing and ceased to grow after 1000 s of thermal annealing. Figure 4 displays both the simulated and measured reflection spectra of the hybrid PTE sample after different thermal annealing treatments under TM-polarized light incidence (θ = 15°). As shown in Figure 4a, the h-LSP mode for *w* = 1.4 μm exhibits stepwise redshifts from λ = 9.50 μm to λ = 9.69 μm as the annealing time increases to 1000 s. This is because the optical refractive index of GST increases during the phase transition from the amorphous to the partially crystalline state.^59–62^ A similar stepwise redshift trend can also be observed for the samples with *w* = 1.7 μm and *w* = 2.0 μm, resulting in a wavelength shift of 0.17 μm and 0.16 μm, respectively.

**Figure 4:**
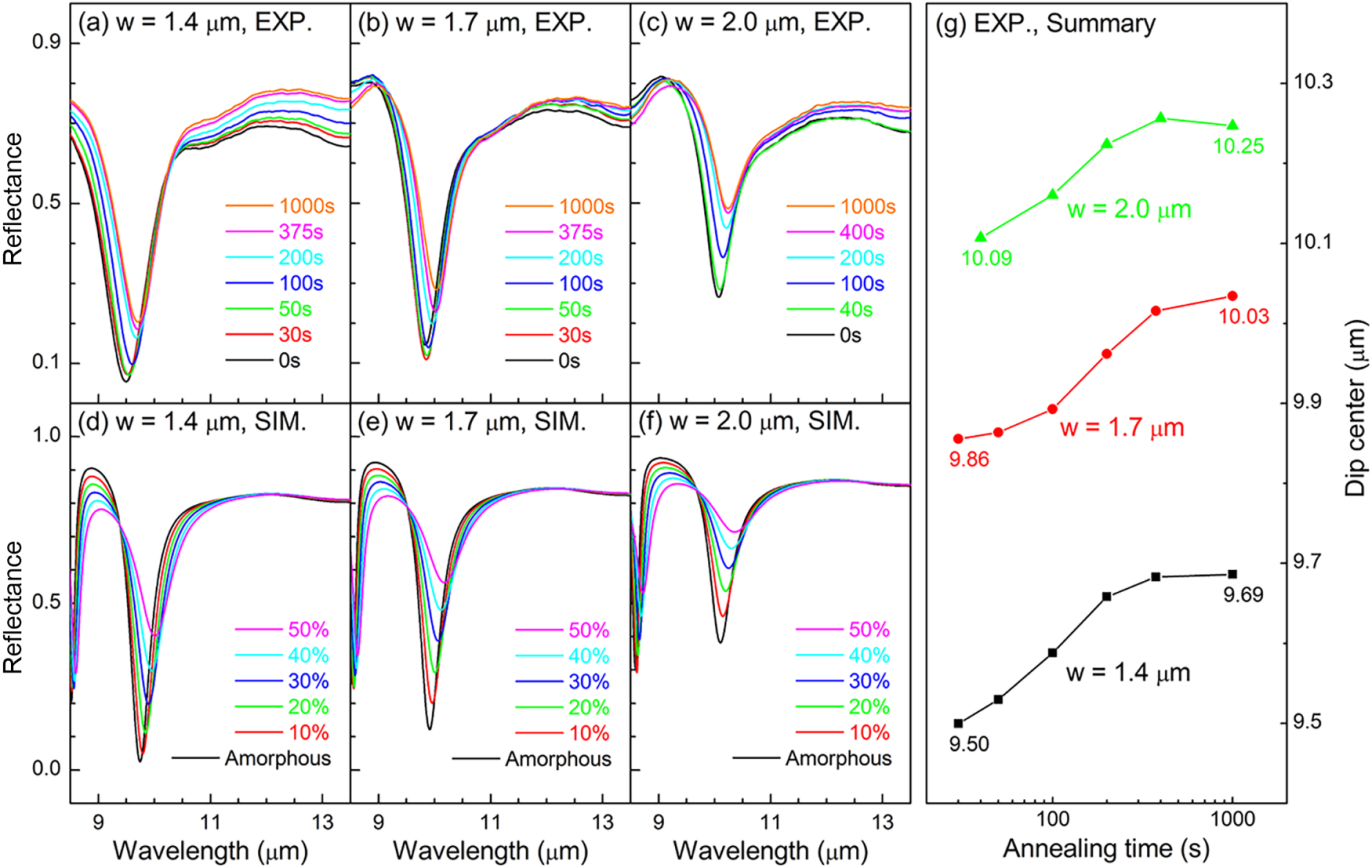
Measured reflectance spectra for the samples with the grating width of (a) 1.4 μm, (b) 1.7 μm, and (c) 2.0 μm under different durations of thermal annealing. (d–f) Simulated spectra for the corresponding samples under the oblique incidence of TM-polarized light (θ = 15°) when the GST layer is transformed from 0% (aGST) to 50 % (semi-cGST). (g) The variation of the measured dip positions of the h-LSP modes as a function of annealing time.

Meanwhile, one can observe that the h-LSP mode gradually becomes broader and shallower as the extinction coefficient of the GST layer increases at higher crystalline fractions. Interestingly, we found that the wavelength variation of the h-LSP mode does not follow a linear relationship with annealing time across the three samples. Figure 4g summarizes the dependence of the measured spectral shift of the h-LSP mode as a function of annealing time among the three samples. We observed a nonuniform spectral shift (slow–fast–slow progress) for our samples: a subtle wavelength shift in the first beginning 50 s, reach an intensive variation between 100 s and 400 s of annealing, and the increasing tendency to drop once the annealing time exceeded 400 s. The phase transition of GST caused by 160 °C thermal annealing reached maximum crystallinity in 1000 s though crystalline percentage might still be far away from the full transition.

To quantitatively describe the effective crystalline fraction of the GST layer under different thermal annealing treatments, we also simulated the reflectance spectra of the hybrid PTE structure using different effective permittivity values for the GST layer to mimic varying degrees of crystallinity, as evaluated by the Lorentz–Lorenz equation. However, one should notice that the numerical model does not consider the regional variation during the heating process. When the GST layer is too thick, partial crystallization of GST may happen.^63^ Therefore, the simulation results can only provide a primitive approximation for the evaluation of the percentage of phase change by considering its average effect. As shown in Figure 4d, the resonant wavelength of the h-LSP mode for the sample with *w* = 1.4 μm is initially at 9.75 μm and gradually redshifts when the crystalline percentage of the GST increases, while maintaining the *Q*-factor of 24.7. To reach the same amount of the spectra shift compared to the measured results with the thermal annealing of 1000 s, we found that it approximately corresponds to the case of 40*%* phase change from the simulations (Figure 4d–f). In addition, the simulated spectra also show broadening and shallower resonances at higher crystallin percentage due to the increasing extinction coefficient of the GST. Table 2 summarizes the simulated and measured reflection spectral shifts for three samples. It can be observed that the spectral shift of the h-LSP mode induced by the GST phase transition remains highly robust across samples with different structural parameters.

**Table 2:** Spectral Shift of the h-LSP Mode in the Reflection Induced by the Phase Transition of GST (Unit: μm)

	*w*	λ_0%_	λ_40%_	λ_0 → 40%_	λ_50%_	λ_0 → 50%_		*w*	λ_0 s_	λ_1000 s_	λ_0→1000 s_
TM, SIM.	1.4	9.75	9.95	0.19	10.00	0.25	TM, EXP.	1.4	9.50	9.69	0.19
TM, SIM.	1.7	9.93	10.12	0.20	10.17	0.25	TM, EXP.	1.7	9.86	10.03	0.17
TM, SIM.	2.0	10.12	10.32	0.20	10.37	0.25	TM, EXP.	2.0	10.09	10.25	0.16

Finally, these samples were placed on a ceramic heating plate and supplied with a constant current source, and their emission spectra were measured at 100 °C. A low temperature of 100 °C was chosen to enable measurement of the emission spectra without triggering phase changes in the GST layer (see the phase stability of GST during continuous emission operation at different operating temperatures and under long-term storage in Sections S6 and S7, respectively).^61,64,65^ The emission temperature was verified by a thermal imaging camera and a thermal couple at the same time. To characterize the h-LSP mode individually, a polarizer was added in front of the FTIR during the thermal emission measurement. Figure 5a–c shows the measured emission spectra for the three samples that undergo the same thermal annealing process displayed in Figure 4. All spectra were calibrated to a blackbody emission at 100 °C. The measured emission spectra of the h-LSP mode exhibit a redshift from λ = 9.74 to 9.86 μm for *w* = 1.4 μm, from 9.79 to 9.94 μm for *w* = 1.7 μm, and from 10.16 to 10.29 μm for *w* = 2.0 μm. Meanwhile, the Q-factor of the emission peaks has an averaged value of 8.70, 12.72, and 15.68 for *w* = 1.4 μm, 1.7 μm, and 2.0 μm, respectively. In addition, as shown in Figure 5d, one can observe the similar nonuniform spectral shift (slow–fast–slow trend) when plotting the emission peak wavelengths as a function of annealing time. Table 3 summarizes the performance of the proposed device and lists state-of-the-art MIR selective thermal emitters based on LSP-related designs. While the LSP modes in traditional MDM structures suffer from strong intrinsic ohmic losses due to direct contact with highly lossy metallic reflectors (*Q*-factor ∼4.35–10), the h-LSP mode in our proposed multilayer structure achieves similarly straightforward spectral selectivity through the variation of the grating width (from 8.87 to 9.86 μm in simulation and from 9.50 to 10.09 μm in experiment) and exhibits a significantly improved *Q*-factor (∼18.63). Meanwhile, the phase transition of the GST layer provides additional fine-tuning of the emission peak, with an average shift of 0.14 μm. This capability compensates for fabrication deviations during the photolithography process by enabling postfabrication adjustment of the emission wavelength through control of the GST crystalline state. Such tunability offers additional flexibility for practical applications after device fabrication.

**Figure 5:**
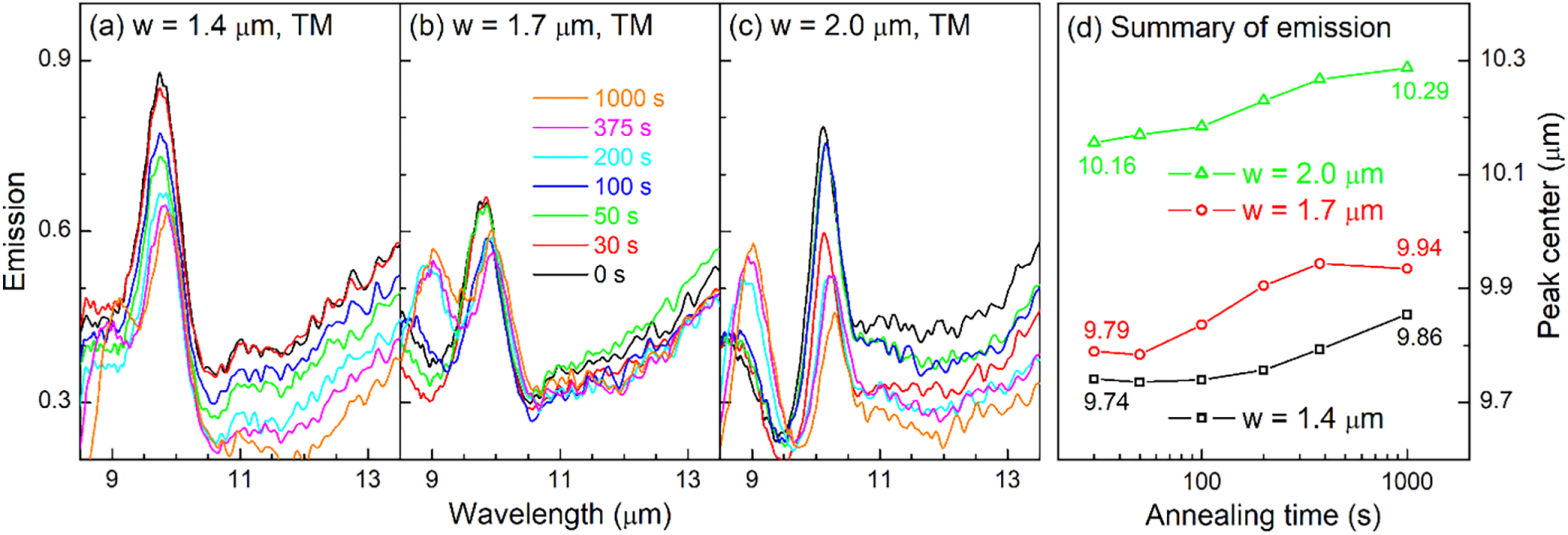
Measured emission spectra for samples with (a) *w* = 1.4 μm, (b) *w* = 1.7 μm, and (c) *w* = 2.0 μm under different durations of thermal annealing. (d) The variation of the measured peak positions of the h-LSP modes as a function of annealing time.

**Table 3:** Performance of State-of-the-Art LSP-Based Thermal Emitters

ref	mechanism	resonance wavelength (μm)	maximum *Q*-factor	tunable range (μm)	source of tunability
^17^	LSP (MDM)	5.8, 8.4	∼10	static	geometric control
^23^	LSP (MDM)	6.7–8.7	8–10	static	geometric control
^24^	LSP (MDM)	1.9, 3.5, 5.9	∼9	static	geometric control
^25^	LSP (MDM)	6.51, 8.70	4–8	6.51–9.33	phase change of GST
^21^	LSP (MDM)	2.58, 2.75, 2.85	∼5	2.58–2.85 (Pixel A)	phase change of GST
				2.75–3.12 (Pixel B)	
				2.85–3.27 (Pixel C)	
^50^	LSP (MDM)	2.5–4.0	∼5	3.35–4.15	phase change of Ge_3_Sb_2_Te_6_
 ^51^	LSP (MDM)	8.43, 9.55	∼8	8.43–9.52 (short axis)	phase change of GST
				9.55–10.78 (long axis)	
^52^	LSP (MDM)	7.8, 8.3	∼6	7.8, 8.3–10.0	phase change of GST
this work (sim.)	h-LSP	9.75–10.12	30.64	9.75 (*w* = 1.4)	geometric control
				9.93 (*w* = 1.7)	
				10.12 (*w* = 2.0)	
				9.75–9.95 (*w* = 1.4)	phase change of GST (λ_0%_ → λ_40%_)
				9.93–10.12 (*w* = 1.7)	
				10.12–10.32 (*w* = 2.0)	
this work (exp.)	h-LSP	9.50–10.09	18.63	9.50 (*w* = 1.4)	geometric control
				9.86 (*w* = 1.7)	
				10.09 (*w* = 2.0)	
				9.50–9.69 (*w* = 1.4)	phase change of GST (*t*_annealing_ = 1000 s)
				9.86–10.03 (*w* = 1.7)	
				10.09–10.25 (*w* = 2.0)	

## 4 Conclusion

In conclusion, we developed a highly reflective, low-loss multilayer back reflector and combined it with a GST layer and top Au gratings to realize high-*Q*, continuously tunable, and small angular dependence thermal emitters. The multilayer back reflector achieves a high reflectance of 90% over the wavelength range of 5.11–9.25 μm and avoids direct contact between the metallic layer and the GST spacer, thereby reducing ohmic loss. Under TM-polarized illumination, the h-LSP mode in the hybrid PTEs exhibits a significant increase in *Q*-factor, reaching 8.81 (24.7), 12.88 (25.1), and 18.63 (23.7) for *w* = 1.4 μm, w = 1.7 μm, and *w* = 2.0 μm, respectively, in the measured (simulated) reflection spectra. In addition, the resonant wavelength of the h-LSP exhibits only a slight blueshift with varying oblique incidence angles. By thermal annealing at 160 °C for varying durations, different levels of crystalline percentage for the GST layer were achieved, and a gradual redshift of the emission peaks is observed: from λ = 9.74 μm to λ = 9.86 μm for *w* = 1.4 μm, from λ = 9.79 μm to λ = 9.94 μm for *w* = 1.7 μm, and from λ = 10.16 μm to λ = 10.29 μm for *w* = 2.0 μm, respectively. This corresponds to an average wavelength shift of 0.14 μm, associated with a 40% GST phase transition. Such a hybrid structure can be fabricated by undergoing a photolithography process once and is compatible with the state-of-the-art complementary metal-oxide-semiconductor (CMOS) technology. Meanwhile, the spectral selectivity achieved through the grating width, combined with fine-tuning via the GST layer phase transition, enables precise control of the emission wavelength, enhancing accuracy in sensing and molecular fingerprint detection.

## Supplementary Material



## Notes

The change of material properties caused by the crystallization of GST, fabrication process of tunable PTEs, simulated and measured reflectance spectra of the multilayer reflectors, effect of the incident angle for different grating widths, evolution of XRD spectra for the tunable PTEs under different thermal annealing durations, phase stability of GST under different emission measurement temperatures, and device stability during long-term storage. (PDF)

## References

[1] Moridani A. K., Zando R., Xie W., Howell I., Watkins J. J., Lee J.-H. (2017). Plasmonic thermal
emitters for dynamically tunable
infrared radiation. Adv. Opt. Mater..

[2] Pfiester N. A., Vandervelde T. E. (2017). Selective emitters for thermophotovoltaic
applications. Phys. Status Solidi A.

[3] Qu Y., Li Q., Cai L., Pan M., Ghosh P., Du K., Qiu M. (2018). Thermal camouflage based on the phase-changing material
GST. Light: Sci. Appl..

[4] Xu R., Lin Y.-S. (2020). Tunable infrared
metamaterial emitter for gas sensing
application. Nanomaterials.

[5] Kang Q., Li D., Guo K., Gao J., Guo Z. (2021). Tunable thermal camouflage
based on GST plasmonic metamaterial. Nanomaterials.

[6] Sun M., Zhang S., Wu D., Han Z. (2024). Highly-efficient gas
sensing using dielectric-based narrow-band thermal emitters operating
in the mid-infrared. Infrared Phys. Technol..

[7] Zhao Z., Wu B., Wang X., Pan Z., Liu Z., Zhang P., Shen X., Nie Q., Dai S., Wang R. (2017). Mid-infrared
supercontinuum covering 2.0–16 *μ*m in
a low-loss telluride single mode fiber. Laser
Photonics Rev..

[8] Jha R. K. (2022). Non-dispersive
infrared gas sensing technology: A review. IEEE
Sens. J..

[9] Liang J.-G., Jiang Y., Wu J.-K., Wang C., von Gratowski S., Gu X., Pan L. (2023). Multiplex-gas detection based on non-dispersive infrared
technique: a review. Sens. Actuators, A.

[10] Chan D. L. C., Soljačić M., Joannopoulos J. (2006). Thermal emission
and design in one-dimensional periodic metallic photonic crystal slabs. Phys. Rev. E.

[11] Rinnerbauer V., Yeng Y. X., Chan W. R., Senkevich J. J., Joannopoulos J. D., Soljačić M., Celanovic I. (2013). High-temperature
stability and selective thermal emission of polycrystalline tantalum
photonic crystals. Opt. Express.

[12] O’Regan B. J., Wang Y., Krauss T. F. (2015). Silicon
photonic crystal thermal
emitter at near-infrared wavelengths. Sci. Rep..

[13] Zhu H., Li Q., Tao C., Hong Y., Xu Z., Shen W., Kaur S., Ghosh P., Qiu M. (2021). Multispectral camouflage
for infrared, visible, lasers and microwave with radiative cooling. Nat. Commun..

[14] Ikeda K., Miyazaki H., Kasaya T., Yamamoto K., Inoue Y., Fujimura K., Kanakugi T., Okada M., Hatade K., Kitagawa S. (2008). Controlled thermal
emission of polarized infrared waves
from arrayed plasmon nanocavities. Appl. Phys.
Lett..

[15] Masuno K., Sawada T., Kumagai S., Sasaki M. (2011). Multiwavelength selective
IR emission using surface plasmon polaritons for gas sensing. IEEE Photonics Technol. Lett..

[16] Liu J., Guler U., Lagutchev A., Kildishev A., Malis O., Boltasseva A., Shalaev V. M. (2015). Quasi-coherent thermal
emitter based on refractory plasmonic materials. Opt. Mater. Express.

[17] Liu X., Tyler T., Starr T., Starr A. F., Jokerst N. M., Padilla W. J. (2011). Taming the blackbody with infrared metamaterials as
selective thermal emitters. Phys. Rev. Lett..

[18] Huang S.-Y., Chen H.-H., Hsiao H.-H., Chang P.-E., Chang Y.-T., Chen C.-H., Jiang Y.-W., Chang H.-C., Lee S.-C. (2012). Triple
peaks plasmonic thermal emitter with selectable wavelength using periodic
block pattern as top layer. IEEE Photonics Technol.
Lett..

[19] Chen H.-H., Hsiao H.-H., Chang H.-C., Huang W.-L., Lee S.-C. (2014). Double
wavelength infrared emission by localized surface plasmonic thermal
emitter. Appl. Phys. Lett..

[20] Hsiao H.-H., Huang C.-H., Xu B.-T., Chen G.-T., Ho P.-W. (2021). Triple
Narrowband midinfrared thermal emitter based on a au grating-assisted
nanoscale germanium/titanium dioxide distributed bragg reflector:
implications for molecular sensing. ACS Appl.
Nano Mater..

[21] Cao T., Zhang X., Dong W., Lu L., Zhou X., Zhuang X., Deng J., Cheng X., Li G., Simpson R. E. (2018). Tuneable
thermal emission using chalcogenide metasurface. Adv. Opt. Mater..

[22] Costantini D., Lefebvre A., Coutrot A.-L., Moldovan-Doyen I., Hugonin J.-P., Boutami S., Marquier F., Benisty H., Greffet J.-J. (2015). Plasmonic metasurface for directional
and frequency-selective
thermal emission. Phys. Rev. Appl..

[23] Dao T. D., Chen K., Ishii S., Ohi A., Nabatame T., Kitajima M., Nagao T. (2015). Infrared perfect absorbers
fabricated
by colloidal mask etching of Al–Al2O3–Al trilayers. ACS Photonics.

[24] Liao C. Y., Wang C.-M., Cheng B. H., Chen Y.-H., Tsai W.-Y., Feng D.-Y., Yeh T. T., Yen T.-J., Tsai D. P. (2016). Quasi-coherent
thermal radiation with multiple resonant plasmonic cavities. Appl. Phys. Lett..

[25] Qu Y., Li Q., Du K., Cai L., Lu J., Qiu M. (2017). Dynamic thermal
emission control based on ultrathin plasmonic metamaterials including
phase-changing material GST. Laser Photonics
Rev..

[26] Auguié B., Fuertes M. C., Angelomé P. C., Abdala N. L., Soler
Illia G. J., Fainstein A. (2014). Tamm plasmon resonance in mesoporous
multilayers: toward a sensing application. ACS
Photonics.

[27] Yang Z.-y., Ishii S., Yokoyama T., Dao T. D., Sun M.-g., Nagao T., Chen K.-p. (2016). Tamm plasmon
selective thermal emitters. Opt. Lett..

[28] Yang Z.-Y., Ishii S., Yokoyama T., Dao T. D., Sun M.-G., Pankin P. S., Timofeev I. V., Nagao T., Chen K.-P. (2017). Narrowband
wavelength selective thermal emitters by confined Tamm plasmon polaritons. ACS Photonics.

[29] Liu X., Li Z., Wen Z., Wu M., Lu J., Chen X., Zhao X., Wang T., Ji R., Zhang Y. (2019). Large-area, lithography-free, narrow-band and highly
directional
thermal emitter. Nanoscale.

[30] Yao K., Ma H., Huang M., Zhao H., Zhao J., Li Y., Dou S., Zhan Y. (2019). Near-perfect selective photonic crystal emitter with
nanoscale layers for daytime radiative cooling. ACS Appl. Nano Mater..

[31] Kaliteevski M., Iorsh I., Brand S., Abram R., Chamberlain J., Kavokin A., Shelykh I. (2007). Tamm plasmon-polaritons: Possible
electromagnetic states at the interface of a metal and a dielectric
Bragg mirror. Phys. Rev. B.

[32] Azzini S., Lheureux G., Symonds C., Benoit J.-M., Senellart P., Lemaitre A., Greffet J.-J., Blanchard C., Sauvan C., Bellessa J. (2016). Generation and spatial control of
hybrid Tamm plasmon/surface plasmon modes. ACS
Photonics.

[33] Aharon O., Abdulhalim I. (2009). Liquid crystal Lyot tunable filter with extended free
spectral range. Opt. Express.

[34] Coles H., Morris S. (2010). Liquid-crystal lasers. Nat. Photonics.

[35] Kumar A., Singh G. (2023). Recent advances and future perspectives
of photoluminescent liquid
crystals and their nanocomposites for emissive displays and other
tunable photonic devices. J. Mol. Liq..

[36] Wu G., Wei X., Gao S., Chen Q., Peng L. (2016). Tunable graphene micro-emitters
with fast temporal response and controllable electron emission. Nat. Commun..

[37] Ghirardini L., Pogna E. A., Soavi G., Tomadin A., Biagioni P., Dal Conte S., Mignuzzi S., De Fazio D., Taniguchi T., Watanabe K. (2021). Tunable broadband light emission from graphene. 2D Mater..

[38] Gutruf P., Zou C., Withayachumnankul W., Bhaskaran M., Sriram S., Fumeaux C. (2016). Mechanically tunable
dielectric resonator
metasurfaces at visible frequencies. ACS Nano.

[39] Bei L., Dennis G. I., Miller H. M., Spaine T. W., Carnahan J. W. (2004). Acousto-optic
tunable filters: fundamentals and applications as applied to chemical
analysis techniques. Prog. Quantum Electron..

[40] Abdollahramezani S., Hemmatyar O., Taghinejad H., Krasnok A., Kiarashinejad Y., Zandehshahvar M., Alù A., Adibi A. (2020). Tunable nanophotonics
enabled by chalcogenide phase-change materials. Nanophotonics.

[41] Long L., Taylor S., Wang L. (2020). Enhanced infrared emission by thermally
switching the excitation of magnetic polariton with scalable microstructured
VO2 metasurfaces. ACS Photonics.

[42] Liang S., Xu F., Li W., Yang W., Cheng S., Yang H., Chen J., Yi Z., Jiang P. (2023). Tunable smart mid infrared
thermal control emitter based on phase change material VO_2_ thin film. Appl. Therm. Eng..

[43] Jiang X., Nong J., Li X., Liao X., Zeng J., Luo S., Zhang Z., Du T., Chen H., He X., Yu Y., Zhang Z., Zhang S., Liu D., Wu J., Yang J. (2025). Laser-Adaptive
Inverse-Design Metamaterials for Durable Regulation
from Visible-Infrared-LiDAR Compatible Camouflage to Optical Limiter. Laser Photonics Rev..

[44] Li X., Liao X., Zeng J., Yi Z., He X., Wu J., Chen H., Zhang Z., Yu Y., Zhang Z., Huang S., Yang J. (2025). Non-volatile tunable
multispectral
compatible infrared camouflage based on the infrared adiation characteristics
of Rosaceae plants. Opto-Electron. Adv..

[45] Delaney M., Zeimpekis I., Lawson D., Hewak D. W., Muskens O. L. (2020). A new family
of ultralow loss reversible phase-change materials for photonic integrated
circuits: Sb_2_S_3_ and Sb_2_Se_3_. Adv. Funct. Mater..

[46] Ling Y.-C., Yoo S. J. B. (2023). tunable nanophotonic metastructures. Nanophotonics.

[47] Tripathi D., Hegde R. S. (2024). Phase change material metasurface
loading enables an
ultrafast all-optically switchable, compact, narrowband free space
optical filter. Opt. Commun..

[48] Du K.-K., Li Q., Lyu Y.-B., Ding J.-C., Lu Y., Cheng Z.-Y., Qiu M. (2017). Control over
emissivity of zero-static-power thermal emitters based
on phase-changing material GST. Light: Sci.
Appl..

[49] Du K., Cai L., Luo H., Lu Y., Tian J., Qu Y., Ghosh P., Lyu Y., Cheng Z., Qiu M., Li Q. (2018). Wavelength-tunable
mid-infrared thermal emitters with a non-volatile
phase changing material. Nanoscale.

[50] Tittl A., Michel A.-K. U., Schäferling M., Yin X., Gholipour B., Cui L., Wuttig M., Taubner T., Neubrech F., Giessen H. (2015). A Switchable
Mid-Infrared Plasmonic Perfect Absorber with Multispectral Thermal
Imaging Capability. Adv. Mater..

[51] Qu Y., Li Q., Cai L., Qiu M. (2018). Polarization switching of thermal
emissions based on plasmonic structures incorporating phase-changing
material Ge_2_Sb_2_Te_5_. Opt. Mater. Express.

[52] Chen L., Sun L., Dong H., Mou N., Zhang Y., Li Q., Jiang X., Zhang L. (2020). Near-field
imaging of the multi-resonant
mode induced broadband tunable metamaterial absorber. RSC Adv..

[53] Zhu H., Luo H., Li Q., Zhao D., Cai L., Du K., Xu Z., Ghosh P., Qiu M. (2018). Tunable narrowband mid-infrared thermal
emitter with a bilayer cavity enhanced Tamm plasmon. Opt. Lett..

[54] Williams C., Hong N., Julian M., Borg S., Kim H. J. (2020). Tunable
mid-wave infrared Fabry-Perot bandpass filters using phase-change
GeSbTe. Opt. Express.

[55] Palik, E. D. Handbook of Optical Constants of Solids; Academic Press, 1998; Vol. 3, pp xvii–xviii.

[56] Chu C. H., Tseng M. L., Chen J., Wu P. C., Chen Y.-H., Wang H.-C., Chen T. Y., Hsieh W. T., Wu H. J., Sun G., Tsai D. P. (2016). Active dielectric metasurface based on phase-change
medium. Laser Photonics Rev..

[57] Raoux S., Jordan-Sweet J. L., Kellock A. J. (2008). Crystallization properties of ultrathin
phase change films. J. Appl. Phys..

[58] Fillot F., Sabbione C. (2020). Nanoscale mechanics of thermally crystallized GST thin
film by in situ x-ray diffraction. J. Appl.
Phys..

[59] Cinkaya H., Ozturk A., Hasekioğlu A.
S. A., Kaya Z. E., Kalem S., CharpinNicolle C., Bourgeois G., Guillaume N., Cyrille M. C., Garrione J. (2021). Structural
properties of Ge-Sb-Te alloys. Solid-State Electron..

[60] Jones R. O. (2024). Phase change
memory materials: Why are alloys of Ge, Sb, and Te the materials of
choice?. Solid State Sci..

[61] Frantz J. A., Myers J. D., Clabeau A., Bekele R. Y., Hong N., Vincenti M. A., Gandolfi M., Sanghera J. S. (2023). Optical constants
of germanium antimony telluride (GST) in amorphous, crystalline, and
intermediate states. Opt. Mater. Express.

[62] Guo P., Sarangan A. M., Agha I. (2019). A review of
germanium-antimony-telluride
phase change materials for non-volatile memories and optical modulators. Appl. Sci..

[63] Kang D., Kim Y., Lee M. (2023). Multispectral
Imaging with a Planar Cavity-Type Metasurface
for Optical Security. ACS Appl. Mater. Interfaces.

[64] Bragaglia V., Jenichen B., Giussani A., Perumal K., Riechert H., Calarco R. (2014). Structural change upon
annealing of amorphous GeSbTe
grown on Si(111). J. Appl. Phys..

[65] Burr G. W., Tchoulfian P., Topuria T., Nyffeler C., Virwani K., Padilla A., Shelby R. M., Eskandari M., Jackson B., Lee B. S. (2012). Observation and modeling of polycrystalline
grain formation in Ge_2_Sb_2_Te_5_. J. Appl. Phys..

