# Supplementary material for: Wavelength-Tunable, Low-Angular Dispersion, and Narrowband Thermal Emitters by Incorporating Ge2Sb2Te5 Layer into Grating-Assisted Multilayered Structures
Source: ACS Appl Mater Interfaces. 2026 Jun 22;18(26):36915–23. doi: 10.1021/acsami.6c06818 (PMC13352512; doi:10.1021/acsami.6c06818)
Supplement: Supplementary file 1 [file am6c06818_si_001.pdf]

Supporting Information

Wavelength-tunable, Low-angular Dispersion, and Narrowband Thermal Emitters by Incorporating $Ge_2Sb_2Te_5$ Layer into Grating-assisted Multilayered Structures

Yuan-Wei Chang,[1] Po-Wei Ho,[2] and Hui-Hsin Hsiao[1,3,*]

[1]Department of Engineering Science and Ocean Engineering, National Taiwan University, Taipei 10617, Taiwan

[2]Institute of Electro-Optical Engineering, National Taiwan Normal University, Taipei 11677, Taiwan

[3]Graduate Institute of Photonics and Optoelectronics, National Taiwan University, Taipei, 10617, Taiwan

[*]E-mail: hhhsiao@ntu.edu.tw

This supplementary information included the following sections:

- The change of material properties caused by the crystallization of GST.
- Fabrication process of tunable PTEs.
- Simulated and measured reflectance spectra of the multilayer reflectors.
- Effect of the incident angle for different grating widths.
- Evolution of XRD spectra for the tunable PTEs under different thermal annealing durations.
- Phase stability of GST under different emission measurement temperatures
- Device stability during long-term storage

S1 The change of material properties caused by the crystallization of GST

Figure S1 shows the refractive index of the GST layer used in our numerical model [1]. The refractive index of amorphous GST (a-GST, n ≈ 3.75) is close to that of Ge (n ≈ 4.02) in the mid-infrared (MIR) wavelength region. After crystallization, the refractive index of crystalline GST (c-GST) increases to approximately 5.3–6, accompanied by a slight increase in the extinction coefficient (*k*). By applying the Lorentz–Lorenz equation, the effective permittivity of GST with different levels of crystallinity was calculated [2].

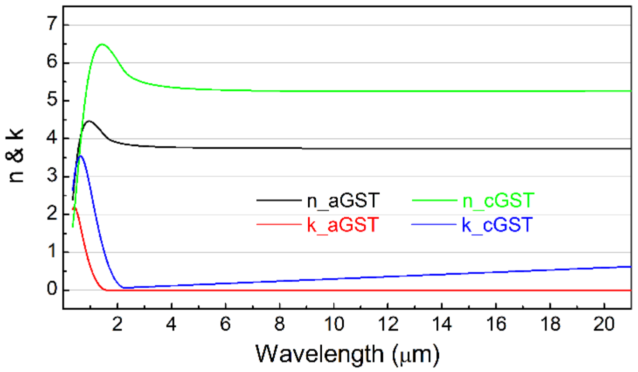

Figure S1: The refractive index of aGST and cGST before and after crystallization.

S2 Fabrication process of tunable PTEs

Figure S2 illustrates the fabrication process of the hybrid PTEs. Figures 3(a)–(d) present the top and cross-sectional SEM images of the fabricated samples. The designed and measured thicknesses of each layer are listed in Table 1.

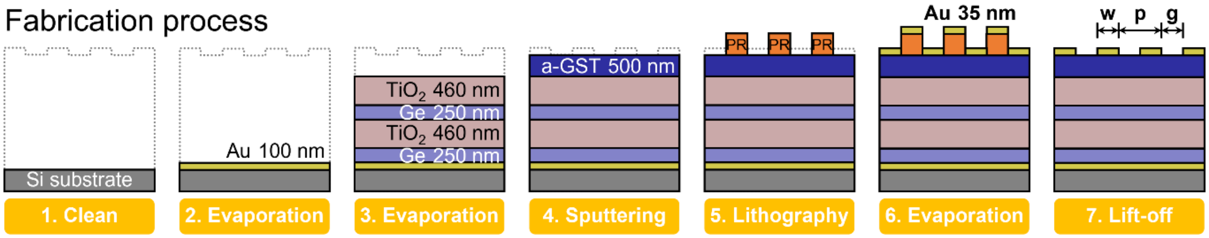

Figure S2: The schematic illustration of fabrication process of tunable PTEs.

S3 Simulated and measured reflectance spectra of the multilayer reflectors

Figure S3 shows the simulated reflection spectra of the multilayer reflectors, consisting of a four-layer Ge/TiO_2 distributed Bragg reflector (DBR) and a bottom Au reflector. The DBR was designed for a central wavelength of 4 µm, corresponding to quarter-wave optical thicknesses of 250 nm for Ge and 460 nm for TiO_2. A clear DBR-like stopband with a reflectance exceeding 0.9 is observed between 5.41 µm and 9.25 µm (black curve). Considering thickness deviations in the deposited films, we further simulated the reflectance spectra using the measured film thicknesses listed in Table 1, which results in a redshift of the stopband to the range of 5.83 µm to 9.67 µm (red curve). In addition, due to film roughness and increased scattering, the measured reflection spectra show a reduced reflectance with a maximum value of 0.82 and exhibit more pronounced degradation at wavelengths below 7 µm (green curve).

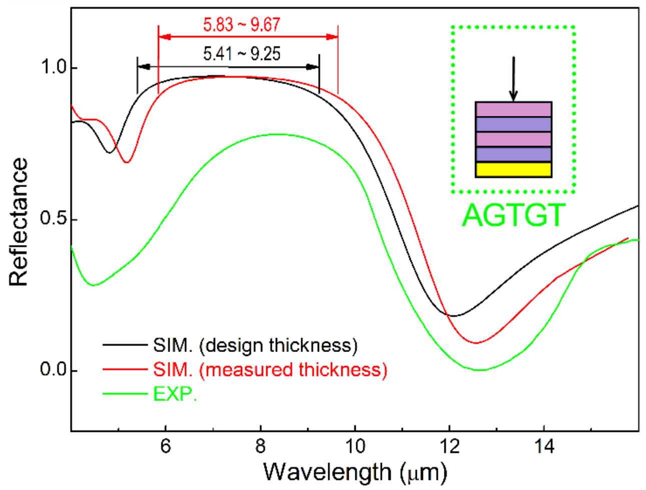

Figure S3: Simulated reflectance spectra of the multilayer reflector for the designed thicknesses (black curve) and the deposited thicknesses extracted from cross-sectional SEM images (red curve), along with the measured reflectance spectra (green curve).

S4 Effect of the incident angle for different grating widths

Figure S4 display the simulated and measured absorption spectra of the tunable PTEs under oblique incidence of TM-polarized light. The simulated spectra for the grating widths of w = 1.45 μm and w = 2.05 μm demonstrate the h-LSP mode exhibits a slightly blueshift when the oblique incident is varied from 0° to 45°. We also measured the absorption spectra of fabricated samples by varying the oblique incident angle from 15° to 60° in 5° increments. To avoid the collision between the reflecting mirrors in our measurement setup, the minimal oblique angle is limited to 15° [3]. As shown in Fig. S4(b) and (d), the measured spectra well aligned with the simulated results, showing small angular dependence of the h-LSP mode.

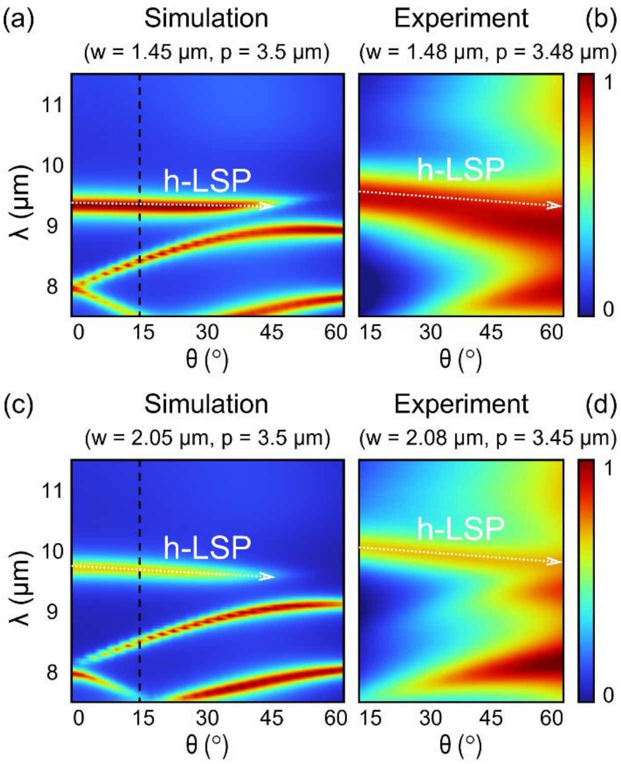

Figure S4: Under oblique incidence of TM-polarized light, (a) the simulated and (b) measured absorption spectra of the tunable PTEs for a grating width of w = 1.45 μm, along with the corresponding (c) simulated and (d) measured absorption spectra for w = 2.05 μm.

To quantitatively analyze the wavelength shift as a function of angle, Figure S5 shows the resonance wavelength versus the oblique incidence angle for three samples with grating widths of 1.45 μm, 1.75 μm, and 2.05 μm, respectively. The measured data for w = 1.45 μm (black solid symbol) exhibit a 0.43 μm blueshift as the oblique incidence angle increases from 15° to 50°, while those for w = 1.75 μm (red solid symbol) and w = 2.05 μm (green solid symbol) show blueshifts of 0.28 μm and 0.31 μm, respectively. On the other hand, the simulated spectra for w = 1.45 μm initially exhibits a blueshift trend, which then changes to a redshift tendency when the incidence angle exceeds 30°. This behavior is attributed to the influence of another

mode at shorter wavelengths that lies in close spectral proximity to the h-LSP mode at larger incidence angles. Such a phenomenon becomes less pronounced when the h-LSP mode is initially excited at longer wavelengths in structures with larger grating widths. For the cases of w = 1.75 μm and w = 2.05 μm, the h-LSP mode exhibits a subtle blueshift as the oblique incidence angle varies from 0° to 50°, resulting in spectral shifts of 0.04 μm and 0.13 μm, respectively.

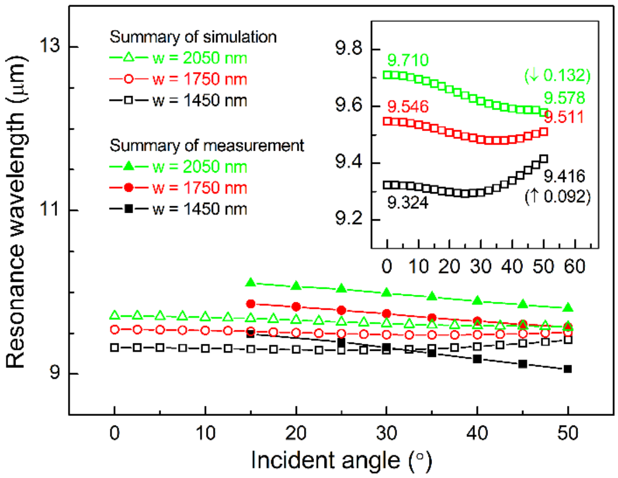

Figure S5: Resonance wavelength as a function of oblique incidence angle for samples with different grating widths. Solid and hollow symbols represent the experimental and simulated results, respectively.

S5 Evolution of XRD spectra for the tunable PTEs under different thermal annealing durations

Figure S6 presents the XRD results of the tunable PTEs under different thermal annealing treatments. The diffraction peaks corresponding to Au (111) and (200), located at 38.1° and 44.4°, respectively, remain present regardless of annealing duration. In contrast, the peak intensities of GST (111), (200), and (220), located at 25.5°, 29.5°, and 42.3°, respectively, increase with annealing time, indicating the formation of crystalline GST (c-GST).

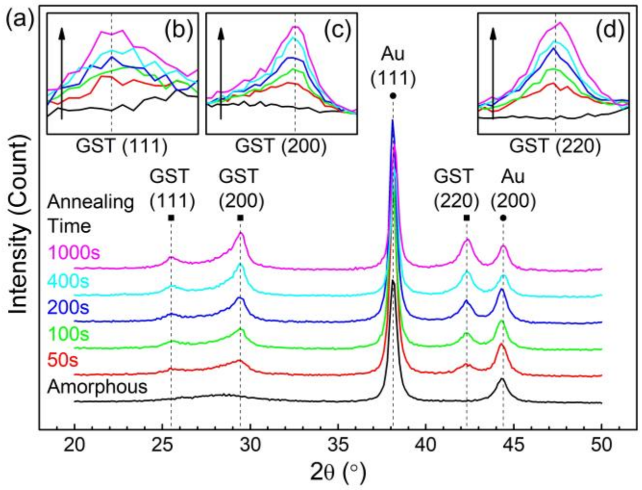

Figure S6: (a) Evolution of the XRD spectra of the tunable PTEs under different thermal annealing durations at 160 °C. Enlarged views of the GST peaks: (b) (111), (c) (200), and (d) (220).

S6 Phase stability of GST under different emission measurement temperatures

To evaluate the performance and stability of the PTEs, we measured the emission spectra at two different temperatures (100 °C and 120 °C) for samples before (i.e., before phase transition) and after thermal treatment at 160 °C for 1000 s (i.e., after phase transition). The device was placed on a ceramic heating stage driven by a constant current source. Once thermal equilibrium was reached, we recorded the emission spectra as a function of measurement time from 0 to 3000 s. Figures S7(a) and S7(b) show the emission spectra measured at 100 °C for samples before and after the phase transition, respectively. To enable a quantitative comparison, we extract the peak wavelength and the full width at half maximum (FWHM) of the emission spectra as a function of measurement time. As shown in Figs. S7(e) and S7(f), the emission peak wavelength remains nearly unchanged for both samples before and after the phase transition, demonstrating the robustness of the emission profile at 100 °C. The slight variation in the FWHM may be attributed to measurement noise.

Next, we repeated the same measurement procedure while increasing the measurement temperature to 120 °C. Figures S7(c) and S7(d) show the emission spectra measured at 120 °C for samples before and after the phase transition, respectively. It can be observed that, for the sample before the phase transition, the emission wavelength gradually redshifts with increasing measurement time, shifting from 10.15 μm to 10.21 μm after 3000 s of testing at 120 °C. In contrast, the emission wavelength remains stable for the sample after the phase transition. This behavior is attributed to the thermal treatment at 160 °C for 1000 s, after which the GST phase transition slows down significantly at this temperature (Figures 4g and 5d). Thus, the relatively

low measurement temperature (120 °C) does not further induce phase changes in the GST. Similarly, the FWHM of the emission spectra shows only slight variation at 120 °C.

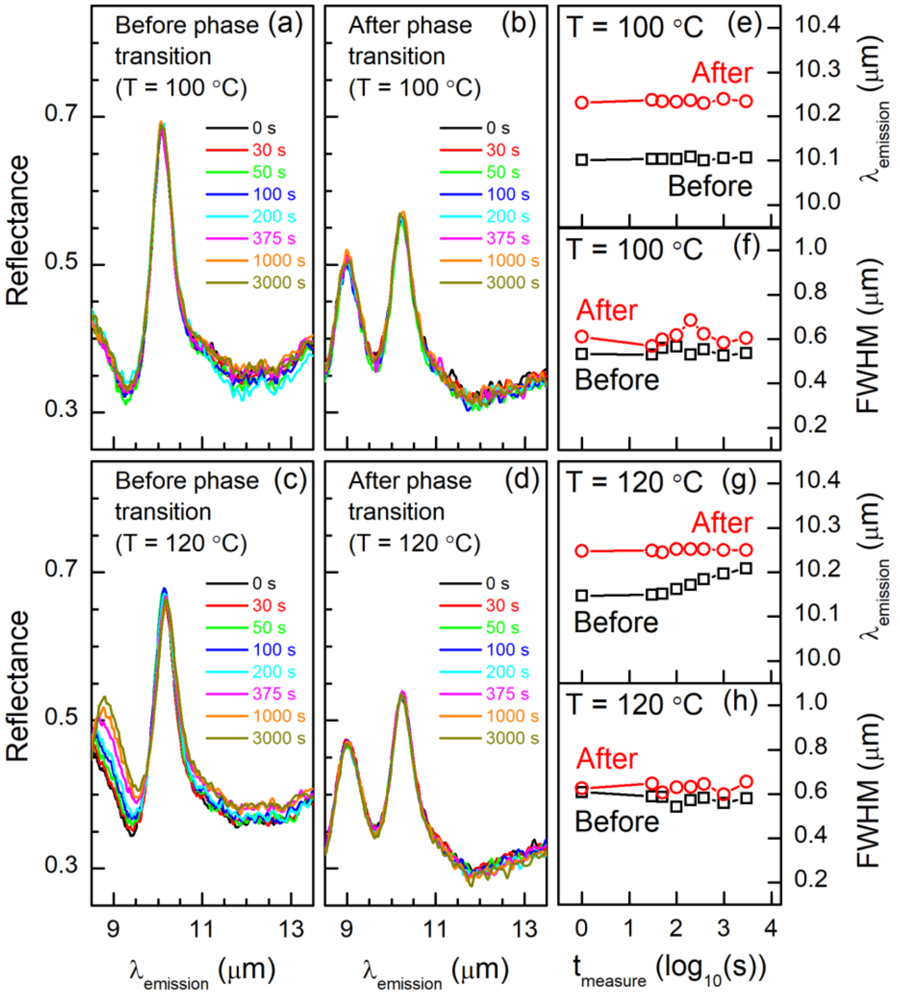

Figure S7: Emission spectra measured at 100 °C for samples (a) before and (b) after the GST phase transition, and at 120 °C for samples (c) before and (d) after the GST phase transition. (e) Peak wavelength and (f) FWHM of the emission spectra measured at 100 °C, and (g) and (h) corresponding peak wavelength and FWHM for measurements at 120 °C.

S7 Device stability during long-term storage

Figure S8 shows the measured reflectance spectrum of the device obtained on June 4, 2025 (black curve), while the same sample was remeasured on May 22, 2026 (red and green curves). It can be observed that the three spectra almost completely overlap. For a quantitative comparison, we extracted the dip wavelengths and FWHMs and plotted them in the inset of Fig. S8. The high consistency of these results confirms the excellent long-term stability of the device under ambient storage conditions.

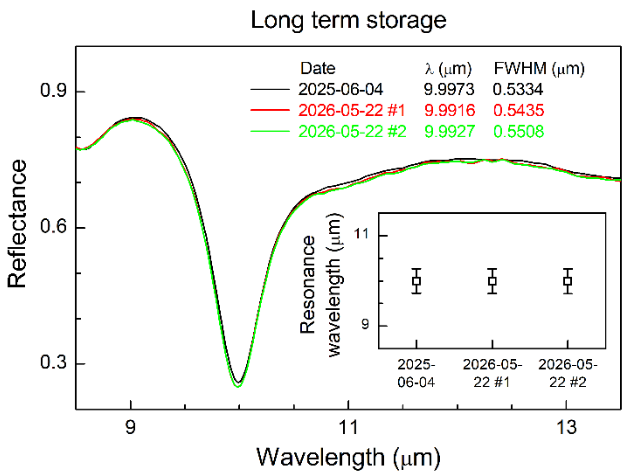

Figure S8: Measured reflectance spectra before (black curve) and after one year of long-term storage under ambient conditions (red and green curves). The extracted dip wavelengths (hollow squares) and FWHMs (vertical bars) are plotted in the inset.

References.

(1) Frantz, J. A.; Myers, J. D.; Clabeau, A.; Bekele, R. Y.; Hong, N.; Vincenti, M. A.; Gandolfi, M.; Sanghera, J. S. Optical constants of germanium antimony telluride (GST)

in amorphous, crystalline, and intermediate states. *Optical Materials Express* **2023**, *13*, 3631–3640.

(2) Chu, C. H.; Tseng, M. L.; Chen, J.; Wu, P. C.; Chen, Y.-H.; Wang, H.-C.; Chen, T.Y.; Hsieh, W. T.; Wu, H. J.; Sun, G.; others Active dielectric metasurface based on phase-change medium. *Laser & Photonics Reviews* **2016**, *10*, 986–994.

(3) Hsiao, H.-H.; Huang, C.-H.; Xu, B.-T.; Chen, G.-T.; Ho, P.-W. Triple Narrowband Mid-Infrared Thermal Emitter Based on a Au Grating-Assisted Nanoscale Germanium/Titanium Dioxide Distributed Bragg Reflector: Implications for Molecular Sensing. *ACS Appl. Nano Mater.* **2021**, *4*, 9344−9352.
